# Antifungal drug susceptibility, molecular basis of resistance to echinocandins and molecular epidemiology of fluconazole resistance among clinical *Candida glabrata* isolates in Kuwait

**DOI:** 10.1038/s41598-020-63240-z

**Published:** 2020-04-10

**Authors:** Zahraa F. Al-Baqsami, Suhail Ahmad, Ziauddin Khan

**Affiliations:** Department of Microbiology, Faculty of Medicine, Kuwait University, Jabriya, Kuwait

**Keywords:** Microbiology, Molecular biology

## Abstract

*Candida glabrata* readily develops resistance to echinocandins. Identification, antifungal susceptibility testing (AST) and resistance mechanism to echinocandins among *C. glabrata* was determined in Kuwait. *C. glabrata* isolates (n = 75) were tested by Vitek2, multiplex PCR and/or PCR-sequencing of rDNA. AST to fluconazole, caspofungin, micafungin and amphotericin B was determined by Etest and to micafungin by broth microdilution (BMD). Mutations in hotspot-1/hotspot-2 of *FKS1*/*FKS2* and *ERG11* were detected by PCR-sequencing. All isolates were identified as *C. glabrata* sensu stricto. Seventy isolates were susceptible and five were resistant to micafungin by Etest and BMD (essential agreement, 93%; categorical agreement, 100%). Three micafungin-resistant isolates were resistant and two were susceptible dose-dependent to caspofungin. Four and one micafungin-resistant isolate contained S663P and ∆659 F mutation, respectively, in hotspot-1 of *FKS2*. Micafungin-resistant isolates were genotypically distinct strains. Only one of 36 fluconazole-resistant isolate contained nonsynonymous *ERG11* mutations. Thirty-four of 36 fluconazole-resistant isolates were genotypically distinct strains. Our data show that micafungin susceptibility reliably identifies echinocandin-resistant isolates and may serve as a surrogate marker for predicting susceptibility/resistance of *C. glabrata* to caspofungin. All micafungin-resistant isolates also harbored a nonsynonymous/deletion mutation in hotspot-1 of *FKS2*. Fingerprinting data showed that echinocandin/fluconazole resistance development in *C. glabrata* is not clonal.

## Introduction

*Candida* spp. are the fourth most common cause of bloodstream infections in hospitalized patients and third common cause of central-line associated invasive infections among intensive care unit (ICU) patients^1–3^. Epidemiological studies have shown that >90% of invasive infections are caused by only five species/species complexes, namely *Candida albicans, Candida glabrata, Candida tropicalis, Candida parapsilosis*, and *Candida krusei*^1–3^. Although *C. albicans* is most commonly isolated from patients with invasive infections and is also the most pathogenic species, infections by non-*albicans Candida* species have increased dramatically in recent years^3–8^. The prophylactic and/or empirical treatment of susceptible immunocompromised/immunosuppressed patients has also resulted in increased prevalence of infections caused by drug-resistant and multidrug-resistant *Candida* species^9–14^. *C. glabrata* has now emerged as the second or third most frequently isolated *Candida* species from patients, particularly from critically ill older (>65 years) patients, with bloodstream and other invasive infections as well as those with vulvovaginal and oral infections^6,14–18^.

*C. glabrata*, a haploid fungal pathogen, is intrinsically less susceptible to azole antifungal drugs and invasive infections are associated with high (~50%) mortality rates, particularly in immunocompromised elderly patients requiring major surgery and neutropenic patients^2,5–7,19,20^. Due to reduced susceptibility of some *Candida* spp. to triazoles, echinocandins were recently promoted as first-line agents for the treatment of invasive *Candida* infections^3^. However, resistance to echinocandins in *Candida* spp. has also appeared in recent years with the highest rate occurring among *C. glabrata* and breakthrough invasive *C. glabrata* infections have been reported in patients on micafungin therapy^14–16,21–24^. Resistance to polyenes is also being reported with increasing frequency in clinical *C. glabrata* isolates^25–29^ and a multidrug-resistant phenotype (resistant to azoles and echinocandins) occurring in ICU and non-ICU settings has also been described in recent years^30,31^.

Acquired resistance to echinocandins in *C. glabrata* makes antifungal susceptibility testing mandatory to guide therapeutic decisions. Echinocandins inhibit cell wall synthesis by binding to their multi-subunit target, 1,3-β-D-glucan synthase complex, encoded by *FKS1, FKS2* and *FKS3* in *C. glabrata* and other *Candida* species^5,19,20^. Studies have shown that clinical echinocandin resistance in *C. glabrata* is due to amino acid substitutions in the hotspot-1 and hotspot-2 regions of the two subunits of 1,3-β-D-glucan synthase complex encoded by *FKS1* and *FKS2* genes^5,19,20^. Detection of mutations in *FKS* genes is now considered as the most accurate method to predict treatment failure even when the patients lack usual risk factors for echinocandin resistance development^20,22–24,32^. This study determined antifungal susceptibility of clinical *C. glabrata* isolates and the molecular basis of resistance to echinocandins by PCR-sequencing of hotspot-1 and hotspot-2 regions of *FKS1* and *FKS2* genes. The *ERG11* was also sequenced from fluconazole-resistant *C. glabrata* isolates. Fingerprinting was carried out to determine whether drug-resistant *C. glabrata* isolates were clonally related.

## Results

### Phenotypic and molecular identification of clinical *C. glabrata* isolates

All 75 isolates initially identified as *C. glabrata* sensu lato by Vitek2 produced purple (mauve) color on CHROMagar Candida and yielded an amplicon of ~212 bp ‘in PCR assay which are characteristic of *C. glabrata* sensu stricto strains. PCR-sequencing of the internally transcribed spacer (ITS) region (including ITS-1–5.8 S rRNA-ITS-2) of rDNA also identified all 51 selected isolates as *C. glabrata* sensu stricto as they exhibited maximum (>99%) identity with reference *C. glabrata* strains ATCC90030 or CBS138. The ITS region sequence data also showed genotypic heterogeneity as 23 different haplotypes (ITSH1 to ITSH23) were detected among 51 *C. glabrata* isolates (16 isolates yielded unique haplotypes while the remaining seven haplotypes were shared among 35 isolates in seven clusters) from Kuwait.

### Antifungal susceptibility profile of *C. glabrata* isolates

The antifungal susceptibility testing (AST) data against micafungin, caspofungin, fluconazole, and amphotericin B by Etest are presented in Table 1. According to EUCAST clinical breakpoints, 70 of 75 (93.3%) isolates were susceptible to micafungin with a modal minimum inhibitory concentration (MIC) value of 0.016 µg/ml while five isolates exhibited resistance as they showed an MIC value >0.125 µg/ml. The MIC distribution for all susceptible isolates was within two twofold dilution steps surrounding the modal MIC. Interestingly, only three of five micafungin-resistant isolates and one micafungin-susceptible isolate were resistant (MIC ≥ 0.5 µg/ml) to caspofungin by Etest (Table 1). Of the remaining 71 isolates, 24 isolates were in the intermediate range (MIC of >0.25 µg/ml but <0.5 µg/ml) (including two micafungin-resistant isolates) while 47 isolates were susceptible to caspofungin. For fluconazole, 39 of 75 (52%) isolates were susceptible dose-dependent (MIC = < 32 µg/ml) while the remaining 36 (48%) isolates yielded MIC values >32 µg/ml and were scored as resistant (Table 1). For amphotericin B, 70 of 75 (93.3%) isolates yielded MIC values ≤1 µg/ml and were categorized as susceptible (or wild-type) while five (6.7%) isolates were resistant (or non-wild-type) as they yielded MIC values >1 µg/ml (Table 1).

**Table 1:** Antifungal susceptibility patterns of clinical *C. glabrata* isolates against various antifungal agents by Etest.

Antifungal	Number of isolates with indicated minimum inhibitory concentration (MIC) in µg/ml																								
drug	≤0.008	0.012	0.016	0.023	0.032	0.047	0.064	0.094	0.125	0.19	0.25	0.38	0.5	0.75	1	1.5	2	3	4	6	8	12	16	24	≥32
**Micafungin**	13	16	28	11	2	0	0	0	**4**	0	**1**	0	0	0	0	0	0	0	0	0	0	0	0	0	0
**Caspofungin**	1	1	1	3	4	2	3	13	7	12	14	10	**1**	**3**	0	0	0	0	0	0	0	0	0	0	0
**Fluconazole**	0	0	0	0	0	0	0	0	1	0	0	1	0	2	0	2	0	2	7	4	6	5	6	3	**36**
**Amphotericin B**	1	1	1	2	2	6	7	10	9	14	6	6	2	1	2	**2**	**1**	**1**	**1**	0	0	0	0	0	0

Isolates with MICs indicative of resistance or reduced susceptibility to antifungal drugs are highlighted in bold. The modal values are underlined.

The AST against micafungin was also carried out by broth microdilution (BMD) method. All five *C. glabrata* isolates resistant to micafungin by Etest were also resistant (MIC > 0.25 µg/ml) by BMD method while the remaining 70 isolates were susceptible (MIC < 0.03 µg/ml). The modal MIC value was 0.007 µg/ml and the MIC distribution for all susceptible isolates was within two twofold dilution steps surrounding the modal MIC. Interestingly, when CLSI clinical breakpoints were considered, 70 isolates were micafungin-susceptible, four were in the intermediate range (but resistant according to EUCAST breakpoints) and one isolate was micafungin-resistant. The correlation between MIC values obtained by Etest and BMD method are presented in Table 2. The data showed that 70 (93.3%) isolates exhibited excellent essential agreement between the two methods as they yielded MIC values that were within 2-fold dilution difference. The remaining five isolates yielded poor essential agreement as they yielded MIC values that differed by >2-fold dilution difference by the two methods. However, the categorical agreement between the two methods was perfect as all five micafungin-resistant isolates by Etest were also resistant to micafungin by BMD and the remaining 70 isolates were susceptible to micafungin by both Etest and BMD method. Interestingly four of five micafungin-resistant isolates exhibited higher MIC values by BMD method (Table 2).

**Table 2:** Correlation between MIC values obtained by Etest and EUCAST broth microdilution (BMD) method during *in vitro* susceptibility testing of 75 *C. glabrata* isolates against micafungin.

		EUCAST MIC (µg/ml)								
		≤0.003	0.007	0.015	0.03	0.06	0.25	1	2	Total
**Etest MIC (µg/ml)**	0.003	1								1
	0.007	5	7							12
	0.015	5	31	6	2					44
	0.03	1*	12							13
	0.06									0
	0.125							1*	3*	4
	0.25						1			1
	1									
	2									
	Total	12	50	6	2	0	1	1	3	75

Etest MIC (µg/ml).

*Isolates with MIC values that differed by >2 fold dilution between Etest and EUCAST BMD method.

Micafungin-resistant isolates are shown in bold.

### Detection of mutations in hotspot-1 and hotspot-2 of *FKS1* and *FKS2* genes

PCR amplification with CgFKS-1F + CgFKS-1R primers yielded an amplicon of ~560 bp from all 75 *C. glabrata* isolates. Similarly, PCR amplification with CgFKS-2F + CgFKS-2R primers yielded an amplicon of ~538 bp from all 75 isolates. The purified amplicons were sequenced with gene and region-specific primers as detailed in ‘Materials and Methods’. Although few synonymous mutations were detected, the translated DNA (amino acid) sequence data for hotspot-1 and hotspot-2 of *FKS1* and hotspot-2 of *FKS2* from all 75 isolates were identical (wild-type) to the sequence from reference *C. glabrata* strain ATCC90030. However, sequence data for hotspot-1 of *FKS2* from only 70 isolates were wild-type while four isolates contained a nonsynonymous (S663P) mutation and one isolate contained a three nucleotide deletion (corresponding to codon F659) (ΔF659). Interestingly, all 70 isolates with wild-type sequence for hotspot-1 of *FKS2* were susceptible to micafungin while four isolates with S663P mutation and one isolate with ∆F659 mutation were resistant to micafungin (Table 3 and Supplementary Table S1). One to five synonymous mutations were also detected within the *FKS2* gene fragment flanking hotspot-1 region in all micafungin-resistant and many micafungin-susceptible isolates.

**Table 3:** Patient's characteristics, clinical source, antifungal drug susceptibility profile and nonsynonymous/deletion mutations in hotspot-1 of *FKS2* among five micafungin-resistant *C. glabrata* isolates.

Patient	Patient’s details		Underlying condition	CFG treatment duration	Clinical source	Isolate no.	Etest MIC (µg/ml) for				Mutation in hotspot-1 of *FKS2*
no.	Gender	Age					MFG	CFG	AMB	FLU	
1	Female	44 years	AML	14 days	Urine	Kw164/15	**0.125**	**0.75**	0.38	**64**	S663P
2	Male	NA	NA	NA	Tracheal secretion	Kw3646/15	**0.125**	0.38	0.19	12	S663P
3	Male	49 years	60% burns	14 days	Urine	Kw458/16	**0.25**	0.38	0.38	8	ΔF659
4	Female	83 years	KTR	14 days	Urine	Kw3554/16	**0.125**	**0.75**	0.094	4	S663P
5	Female	74 years	CKD	14 days	Urine	Kw2138/17	**0.125**	**0.75**	0.094	24	S663P

MIC, minimum inhibitory concentration; CFG, caspofungin; MFG, micafungin; AMB, amphotericin B; FLU, fluconazole; AML, acute myeloid leukemia; KTR, kidney transplant recipient; CKD, chronic kidney disease; NA, not available.

MIC values indicative of resistance to antifungal drugs are shown in bold.

The results of AST for the four antifungal drugs and mutations in *FKS* genes are summarized in Table 3. Only three (Kw164/15, Kw3554/16 and Kw2138/17) micafungin-resistant isolates with mutations in hotspot-1 of *FKS2* exhibited cross-resistance to caspofungin while the remaining two isolates (Kw3646/15 and Kw458/16) with mutations in hotspot-1 of *FKS2* exhibited intermediate susceptibility (MIC = 0.38 µg/ml) to caspofungin by Etest (Table 3). Furthermore, one caspofungin-resistant isolate (Kw330/15) by Etest was susceptible to micafungin by both Etest and BMD method and contained wild-type sequences of hotspot-1 and hotspot-2 of *FKS1* and *FKS2*. Only one of five micafungin resistant *C. glabrata* isolate (Kw164/15) exhibited cross resistance to fluconazole and thus exhibited multidrug-resistant phenotype while all five isolates were susceptible (wild-type) to amphotericin B (MIC < 1 µg/ml) (Table 3). Clinical details and history of previous exposure to echinocandins were available for four patients yielding micafungin-resistant *C. glabrata* isolates with mutations at F659 or S663 in hotspot-1 of *FKS2*. Three patients were females including two elderly patients (>74 years). Four patients had received caspofungin for 14 days as treatment or prophylaxis and *C. glabrata* strains were isolated from urine samples from all four patients (Table 3). The information regarding the isolation of *C. glabrata* from invasive sites from these four patients and the outcome were not available.

**Figure 1:**
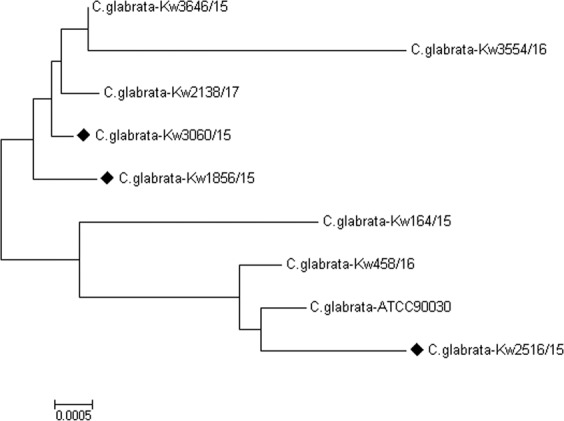
Neighbor-Joining phylogenetic tree based on Maximum Composite Likelihood of DNA sequence data for hotspot-1 and hotspot-2 of *FKS1* and *FKS2* genes together with ITS region of rDNA for five micafungin-resistant *C. glabrata* isolates. Three micafungin-susceptible isolates (**♦)** and reference strain (ATCC90030) of *C. glabrata* were included for comparison purpose.

The phylogenetic relationship among five micafungin-resistant isolates with mutations in hotspot-1 of *FKS2*, three selected micafungin-susceptible isolates and reference *C. glabrata* strain ATCC90030 was also determined from concatenated DNA sequence data for hotspot-1 and hotspot-2 of *FKS1* and *FKS2* and the ITS region of rDNA. The data showed that all five micafungin-resistant isolates were genotypically distinct strains (Fig. 1).

### Analysis of *ERG11* gene sequences of *C. glabrata* isolates

**Figure 2:**
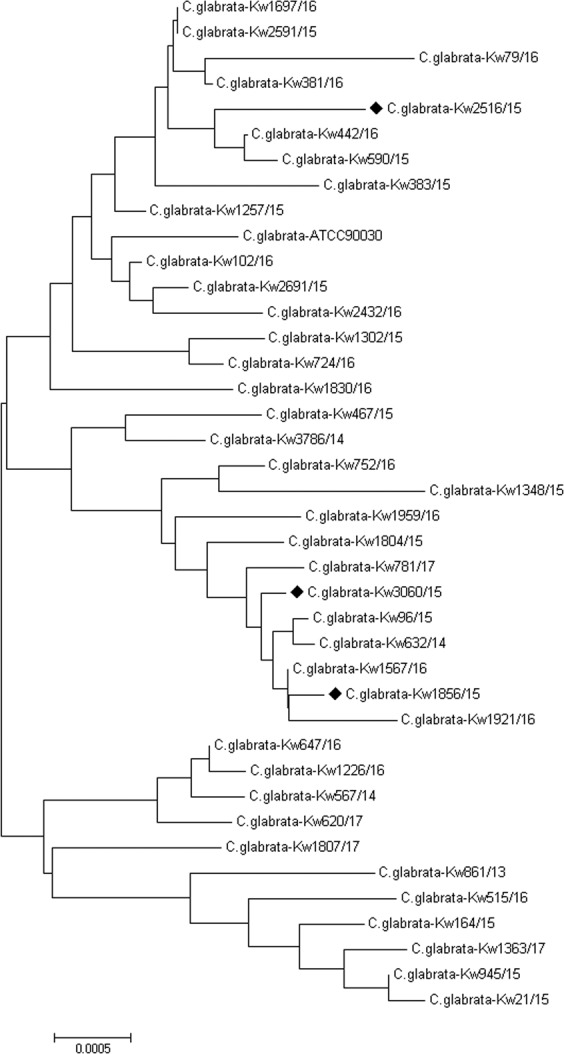
Neighbor-Joining phylogenetic tree based on Maximum Composite Likelihood of concatenated sequence of *ERG11*, ITS region of rDNA, and extended sequences of hotspot-1 and hotspot-2 of *FKS1* and *FKS2* genes for 36 fluconazole-resistant *C. glabrata* isolates. Three fluconazole-susceptible *C. glabrata* isolates (**♦)**, in addition to *C. glabrata* ATCC90030 reference strain were included for comparison purpose.

The *ERG11* gene was amplified as two overlapping fragments and both strands were sequenced from all 36 fluconazole-resistant and three susceptible dose-dependent *C. glabrata* strains. Although few synonymous mutations within the coding region of *ERG11* and/or insertion/deletion/single nucleotide polymorphisms in the non-coding regions were detected (compared to *C. glabrata* ATCC90030), no nonsynonymous mutation was detected in 35 fluconazole-resistant and three susceptible dose-dependent isolates. However, two nonsynonymous mutations (Y141H + L381M) were detected in one fluconazole-resistant isolate (Kw861/13). Isolate Kw861/13 was also sequenced earlier for *ERG11* as part of another study and thus revealed the same mutations described in the previous study^29^. The *ERG11* sequence variations observed in this study were used to study molecular epidemiology of fluconazole-resistance in Kuwait by combining *ERG11* data with data from other loci. Concatenated sequence data comprising *ERG11*, ITS region of rDNA, extended hotspot-1 and hotspot-2 of *FKS1* and *FKS2* gene regions were aligned and the Neighbor-Joining phylogenetic tree is shown in Fig. 2. The data showed that 34 of 36 fluconazole-resistant *C. glabrata* isolates in Kuwait were unique strains while only two fluconazole-resistant isolates shared the same genotype.

## Discussion

All 75 isolates, including 66 isolates collected during 2014–2017, used in this study were identified as *C. glabrata* sensu stricto by a combination of phenotypic and molecular methods. PCR-sequencing of ITS region of rDNA identified 23 different haplotypes among 51 isolates including 16 isolates with unique haplotypes. The data are consistent with a recent study showing that the ITS region of rDNA varies considerably among *C. glabrata* strains^33^. None of the clinical isolate produced creamy white growth on CHROMagar Candida or yielded an amplicon of ~299 bp which is characteristic of *C. bracarensis* strains or an amplicon of ~411 bp which is characteristic of *C. nivariensis* strains in PCR assay^33^.

The AST data by Etest and BMD method identified 70 (93.3%) isolates as susceptible and five isolates as resistant to micafungin. The data also showed that 70 (93.3%) isolates exhibited excellent essential agreement as they yielded MIC values within 2-fold dilution difference by the two methods while only five isolates yielded poor essential agreement. However, the categorical agreement between the two methods was perfect as 70 and five isolates were scored as micafungin-susceptible and micafungin-resistant, respectively, by both methods. Espinel-Ingroff *et al*.^34^, reported an essential agreement of 95% and a categorical agreement of 97% between Etest and CLSI BMD method while Marcos-Zambrano *et al*.^35^, reported an essential agreement of 90% and a categorical agreement of >90% between Etest and EUCAST BMD method. Bougnoux *et al*.^36^, in a recent study based on 933 *Candida* species isolates (including 152 *C. glabrata* isolates) reported an essential agreement of 98.5% and a categorical agreement of 98.2% between Etest and EUCAST BMD method. Similar to these studies, our data also support that Etest is an easy and reliable method for routine AST of clinical *C. glabrata* isolates to micafungin.

Since *FKS1* and *FKS2* genes are homologous, a single amplification primer pair was carefully designed for PCR amplification of hotspot-1 regions of *FKS1* and *FKS2* genes but the sequence of both strands for each gene was obtained by using gene-specific sequencing primers as described under ‘Materials and Methods’. Similarly, hotspot-2 regions of *FKS1* and *FKS2* were also amplified by using another common amplification primer pair and the amplicons were again sequenced by using gene-specific sequencing primers. This novel approach was highly efficient and cost effective as it reduced the work-load and material requirement for PCR amplification reactions and purification of amplicons by 50%.

Although a few synonymous mutations were detected, the amino acid sequence data for hotspot-1 and hotspot-2 of *FKS1* and hotspot-2 of *FKS2* from all 75 isolates were identical (wild-type) to the sequence from reference *C. glabrata* strain ATCC90030. However, amino acid sequence data for hotspot-1 of *FKS2* from only 70 isolates which were susceptible to micafungin were wild-type. Of the five micafungin-resistant isolates, four isolates contained a nonsynonymous (S663P) mutation and one isolate contained a three nucleotide deletion (corresponding to F659; ΔF659) in hotspot-1 of *FKS2*. Thus all five micafungin-resistant isolates from Kuwait harbored a nonsynonymous or deletion mutation in hotspot-1 of *FKS2* only. Our data are consistent with other reports showing that mutations in hotspot-1 of *FKS2* occur more frequently (particularly at F659 and S663) than mutations in hotspot-1 of *FKS1* in echinocandin-resistant *C. glabrata* isolates from diverse geographical locations with S663P mutation occurring more frequently^14,22,37–41^. Similar to another previous study^37^, all five micafungin-resistant *C. glabrata* isolates from Kuwait were also genotypically distinct strains.

Previous studies have shown that S663P and ΔF659 mutations in hotspot-1 of *FKS2* reduce echinocandin sensitivity in mutant 1,3-β-D-glucan synthase and the mutant enzyme exhibits reduced catalytic efficiency relative to wild-type enzyme^42^. Clinical significance of ΔF659 mutation in hotspot-1 of *FKS2* has also been shown in few studies. Lewis *et al*.^32^, reported clinical and microbiological failure in a patient with candidemia due to *C. glabrata*. The initial isolate before therapy was susceptible to micafungin and carried wild-type sequence for *FKS* genes while the isolate obtained after eight days of therapy was resistant to micafungin and contained ΔF659 mutation in hotspot-1 of *FKS2*. Saraya *et al*.^23^, also reported a fatal case of fungemia in a patient due to *C. glabrata*. Resistance to micafungin developed during therapy and the resistant strain contained ΔF659 mutation in hotspot-1 of *FKS2*. Mutations in hotspot-2 of *FKS2* occur rarely while mutations in hotspot-2 of *FKS1*, to the best of our knowledge, have not been reported so far^14,22,37–42^.

The history of previous exposure to echinocandins was available for four of five patients yielding micafungin-resistant *C. glabrata* isolates with mutations at F659 or S663 in hotspot-1 of *FKS2*. Three patients were females including two elderly patients (>74 years). All four patients had received caspofungin for 14 days as treatment or prophylaxis and *C. glabrata* strains were isolated from urine samples from all four patients. Whether *C. glabrata* was also recovered from invasive sites from these four patients was not known. Our results thus confirm previous findings that resistance to echinocandins and *FKS* mutations mainly arise in *Candida* species (including *C. glabrata*) as a result of previous exposure to these drugs which could be as short as 8–13 days^14,22–24,37,39,43^. The data also show that urinary tract provides a favorable niche for easy development of resistance not only to amphotericin B^29^ as was shown recently but also to echinocandins as reported in this study.

Three of five micafungin-resistant isolates were also resistant while two isolates exhibited intermediate resistance to caspofungin by Etest. Cross-resistance among echinocandins has been observed in several studies as they share the same mechanism of action^19,20,42^. In a recent study based on a global collection of invasive *Candida* species isolates collected over two decades (1997 to 2016), 2.2%, 3.5% and 1.7% of *C. glabrata* isolates were resistant to anidulafungin, caspofungin and micafungin, respectively^14^. Surprisingly resistance to micafungin was not detected in *C. glabrata* isolates from Latin America, possibly reflecting the association of resistance development with specific genotypes^14^. As stated above, all five micafungin-resistant isolates (including two isolates with intermediate resistance to caspofungin) harbored a mutation in hotspot-1 of *FKS2*, however, a caspofungin-resistant isolate that was susceptible to micafungin contained wild-type *FKS* sequences in our study. An additional 22 isolates exhibited intermediate resistance to caspofungin but were susceptible to micafungin and contained wild-type sequences for *FKS* genes. The *FKS* mutations are now regarded as a better predictor of non-susceptibility of *C. glabrata* to echinocandins and poor response to treatment^22–24,32,44–46^. Taken together, our data suggest that micafungin can serve as an acceptable surrogate marker for the prediction of susceptibility or resistance of *Candida* species to caspofungin. Our results agree with data reported in few other studies. A previous study involving a large collection (n = 3674) of clinical isolates of eight *Candida* species concluded that micafungin serves as an acceptable surrogate marker for the prediction of susceptibility and resistance of *Candida* spp. to caspofungin^47^. Other studies have also made similar observations due to lack of reproducibility of caspofungin MIC test results or due to high degree of caspofungin MIC variability during both, intra- and interlaboratory testing^48,49^.

Since clinical *Candida* species isolates show wide variations in the MIC values for echinocandins, particularly caspofungin, it has been suggested to use epidemiological cutoff values (ECVs) instead of clinical break points to identify drug-resistant strains^50,51^. Although the number of *C. glabrata* isolates analyzed in this study is small compared to other studies that have used method-dependent ECVs to define *C. glabrata* isolates with reduced susceptibility to echinocandins, we also determined the ECVs for micafungin among our collection of *C. glabrata* isolates. Interestingly all 70 isolates lacking *FKS* mutations yielded ECVs of 0.03 µg/ml by both Etest and BMD method to define wild-type organisms. Consistent with previously defined limits, the distribution of MIC values covered only two twofold dilution steps surrounding the modal MICs^50–52^. The data further support that *FKS* mutations are a better predictor of non-susceptibility of *C. glabrata* to echinocandins^22–24,32,44–46^.

Only one micafungin resistant *C. glabrata* isolate exhibited cross resistance to fluconazole and thus exhibited multidrug-resistant phenotype. On the other hand, all five micafungin resistant *C. glabrata* isolates were susceptible to amphotericin B. In the United States, the rate of resistance of *C. glabrata* to echinocandins has been increasing steadily and ~9% of fluconazole-resistant bloodstream isolates were also resistant to echinocandins^14,22,30,39^. Emergence of resistance to echinocandins in *C. glabrata* and increasing reports of multidrug resistance to azoles, echinocandins and amphotericin B is a worrisome development as it severely limits the choice of antifungal drugs for the treatment of invasive *C. glabrata* infections^13,14,20,30,31^. In this context, multidrug resistance detection in only one *C. glabrata* isolate in Kuwait is encouraging, however, continued surveillance studies are needed to provide accurate estimates of trends in antifungal resistance and their impact on treatment outcome.

*C. glabrata* isolates analyzed in this study included four isolates that exhibited reduced susceptibility to amphotericin B that were analyzed recently for molecular resistance mechanisms^29^. One isolate (Kw861/13) cross-resistant to fluconazole contained two (Y141H + L381M) nonsynonymous mutations which abrogated the function of *ERG11*, accumulated lanosterol and conferred resistance to fluconazole^29^. *C. glabrata* isolates analyzed in this study included 35 other strains with reduced susceptibility to fluconazole. Since non-synonymous mutations in *ERG11* have rarely been reported in fluconazole-resistant *C. glabrata*^29,53^, all 36 fluconazole-resistant and three selected fluconazole-susceptible isolates were analyzed to see if additional fluconazole-resistant strains from Kuwait also contain nonsynonymous/nonsense mutations in *ERG11*.

PCR-sequencing of *ERG11* did not detect any nonsynonymous/deletion mutation in the three susceptible dose-dependent isolates or the remaining 35 fluconazole-resistant isolates analyzed in this study. However, several synonymous mutations within the coding region of *ERG11* and/or insertion/deletion/single nucleotide polymorphisms in the non-coding regions were detected in many isolates. Since in addition to *ERG11*, DNA sequence data for hotspot-1 and hotspot-2 regions of *FKS1* and *FKS2* and ITS region of rDNA were also available for all 36 fluconazole-resistant *C. glabrata* isolates, concatenated sequence data were used for determining genetic relatedness among fluconazole-resistant *C. glabrata* isolates in Kuwait. The phylogenetic tree generated from the combined data sets showed that only two isolates clustered together while 34 isolates were genotypically distinct strains. Thus vast majority of fluconazole-resistant *C. glabrata* strains in Kuwait were genotypically distinct strains implying independent origin of fluconazole resistance development in our isolates. Molecular epidemiology of fluconazole-resistant *C. glabrata* strains has been rarely studied. One study showed that MALDI TOF MS data can be used to classify *C. glabrata* strains according to their fluconazole susceptibility profile^54^. Hou *et al*.^55^, performed molecular fingerprinting of 411 *C. glabrata* isolates (including 68 fluconazole-resistant strains) from China by six-loci-based multilocus sequence typing (MLST) and six-polymorphic markers-based microsatellite typing (MT). Based on MLST, only 35 sequence types (STs) were identified among 273* C. glabrata* isolates and most of the 68 fluconazole-resistant strains clustered into a single ST (ST7). Although MT analysis was more discriminatory as 79 genotypes were identified among 411 *C. glabrata* isolates, 125 (30.4%) and 51 (12.4%) isolates clustered in T25 and T31 types, respectively. Furthermore, T25 and T31 were also the predominant genotypes in fluconazole-resistant isolates^55^. Thus our multiple gene loci-based fingerprinting approach appears to be more discriminatory than MLST or MT analyses for molecular fingerprinting of fluconazole-resistant *C. glabrata* strains.

Our study has few limitations. The AST against fluconazole, caspofungin and amphotericin B was performed only by Etest and not by the reference BMD method. The molecular basis of resistance to fluconazole was investigated only by analysis of *ERG11* while other resistance-conferring mechanisms such as sequence analyses of the transcription factor *PDR1* for gain-of-function mutations were not investigated due to lack of funds.

In conclusion, all 75 clinical isolates used in this study were identified as *C. glabrata* sensu stricto by a combination of phenotypic and molecular methods. Five of 75 *C. glabrata* isolates were resistant to micafungin by both, Etest and the reference BMD method. Only three of these five isolates were also resistant to caspofungin while two showed intermediate resistance. All micafungin-resistant isolates harbored a nonsynonymous or deletion mutation in hotspot-1 of *FKS2* gene and were genotypically distinct strains. All micafungin-susceptible isolates were wild-type for hotspot-1 and hotspot-2 regions of *FKS1* and *FKS2* genes. Multidrug resistance (resistance to fluconazole and echinocandins) was observed in only one isolate. Micafungin clinical breakpoints by EUCAST were more reliable than CLSI breakpoints in discriminating echinocandin-resistant *C. glabrata* isolates from wild-type isolates and micafungin may also serve as a surrogate marker for predicting the susceptibility or resistance of *C. glabrata* to caspofungin. Amphotericin B resistance was detected in four of 75 (5%) *C. glabrata* isolates while fluconazole resistance was detected in 36 of 75 (48%) isolates. Only one *C. glabrata* isolate contained nonsynonymous mutations in *ERG11*. Molecular fingerprinting of fluconazole-resistant isolates showed that 34 isolates were unique strains suggesting that resistance development in *C. glabrata* to fluconazole in Kuwait is not clonal.

## Materials and Methods

### Yeast strains, growth conditions and phenotypic and molecular identification

Reference strains of *C. glabrata* (ATCC90030 and CBS138), *C. albicans* (ATCC90098), *C. parapsilosis* (ATCC22019) and *C. krusei* (ATCC6258) were used during this study. A total of 75 *C. glabrata* isolates originating from urogenital tract (n = 29), respiratory tract (n = 20), bloodstream (n = 12), ascitic/cavitary fluid (n = 3), wound (n = 3), skin (n = 2) and other miscellaneous (peritoneal dialysis fluid, percutaneous endoscopic gastrostomy site, rectum, bed sore, pus, and from an unknown site) sites (n = 6) collected during 2007–2017 and maintained in the Fungal Culture Collection of Mycology Reference Laboratory (Department of Microbiology, Faculty of Medicine, Kuwait University) were used. The isolates were cultured from clinical specimens taken from patients after obtaining verbal consent as part of routine patient care and diagnostic work-up at nine different hospitals across Kuwait. The study did not involve direct contact with patients and the results are reported on deidentified samples without revealing patient identity. The study was approved by the Ethical Committee of Health Sciences Center, Kuwait University (approval letter VDR/EC/30 dated April 6, 2017) and all the methods and investigations were performed in accordance with their guidelines and regulations. The need for informed consent was waived by Health Sciences Center Ethical Committee. The blood specimens were cultured in Bact T/Alert Blood Culture System (BD Diagnostics, Sparks, MD) while other specimens were cultured on Sabouraud dextrose agar (SDA) supplemented with chloramphenicol (50 mg/L) as described previously^56^. The bloodstream isolates were also sub-cultured on SDA with/without additional supplements, as described previously^28^.

The isolates were initially identified as *C. glabrata* sensu lato by Vitek2 yeast identification system (bioMerieux, Marcy-lEtoile, France). All isolates were tested by growth on CHROMagar Candida (Becton Dickinson, Bootle, UK) for phenotypic identification and the results were interpreted according to manufacturer’s instructions and as described previously^57^. The genomic DNA from the isolates was extracted by the rapid boiling method using Chelex-100 or by using Gentra Puregene Yeast DNA extraction kit (Qiagen, Hilden Germany) used according to kit instructions and as described previously^58^. Molecular identification was performed by PCR amplification of internal transcribed spacer (ITS) region of rDNA by using mCGLF, mCNIF, mCBRF and mCGCR primers and detection of amplicons by agarose gel electrophoresis, as described previously^33^. The identity of 51 selected isolates was also confirmed by sequencing of ITS region of rDNA by using panfungal primers, as described previously^59^. BLAST searches (http://blast.ncbi.nlm.nih.gov/Blast.cgi?) were performed and >99% sequence identity was used for species identification^60^.

### Antifungal susceptibility testing

The *in vitro* AST of *C. glabrata* isolates to fluconazole, amphotericin B, caspofungin and micafungin was performed by Etest (bioMérieux SA, Marcy-l’-Etoile, France) in accordance with the manufacturer’s instructions and as described previously^61^. Reference strains of *C. parapsilosis* (ATCC22019) and *C. albicans* (ATCC90028) were used for quality control. The European Committee on Antimicrobial Susceptibility Testing (EUCAST) clinical breakpoints version 9.0 were followed to determine the susceptibility of the isolates as follows: fluconazole; <32 µg/ml, susceptible dose dependent; >64 µg/ml, resistant; amphotericin B; <1 µg/ml, susceptible; >1 µg/ml, resistant; micafungin; ≤0.03 µg/ml, susceptible; >0.03 µg/ml, resistant. However, the Clinical and Laboratory Standards Institute (CLSI) breakpoints were followed to determine the susceptibility of the isolates against caspofungin (EUCAST has not established breakpoints for *Candida* spp. due to high variability in MIC values) and were as follows: ≤0.12 µg/ml, susceptible; 0.25 µg/ml to <0.5 µg/ml, intermediate; ≥0.5 µg/ml, resistant. Quality control was ensured by testing *C. krusei* (ATCC6258) as recommended by EUCAST^62–64^.

The AST of *C. glabrata* isolates to micafungin was also determined by reference BMD method in 96-well tissue culture plate by following the protocol described in the EUCAST Definitive document 7.3.1. The MIC was determined as the drug concentration that yielded ≥50% growth inhibition compared to drug-free control and *C. krusei* (ATCC6258) was used as quality control^62^.

### PCR-sequencing of hotspot-1 and hotspot-2 regions of *FKS1* and *FKS2* genes

The hotspot-1 of *FKS1* and *FKS2* genes was amplified by using a common forward (CgFKS-1F, 5'-ATGCCATTRGGTGGTCTKTTCAC-3') and reverse (CgFKS-1R, 5'-ATRGCAAGYAAATGTTCTCTGTACA-3) primer pair. Similarly, hotspot-2 of both *FKS1* and *FKS2* genes was amplified by using another common forward (CgFKS-2F, 5'-GTGAACAAATGTTGTCCCGTGA-3') and reverse (CgFKS-2R, 5'-GCAAATCTGGAGTAYAAAATKGAGA-3') primer pair. Other PCR reaction and cycling conditions were same as described previously^24^. PCR amplicons were purified and both strands were sequenced for hotspot-1 and hotspot-2 of *FKS1* and *FKS2*. Sequencing reactions with amplicons obtained with CgFKS-1F + CgFKS-1R primers for hotspot-1 of *FKS1* were carried out by using CgFKS1-1FS (5'-AAAGTCTACCAGACGTTACGTC-3') or CgFKS1-1RS (5'-GGAGTCAAAATAGAAATACCCAAG-3') primer and for hotspot-1 of *FKS*2 by using CgFKS2-1FS (5'-CAAAAATCAAGTAGAAGATATGTT-3') or CgFKS2-1RS (5'-AGGAGTTAAGATGGAAATACCTAGA-3') primer. Similarly, sequencing reactions for hotspot-2 of *FKS1* were carried out by using CgFKS1-2FS (5'-AGGTACACAACTTCCAATTGA-3') or CgFKS1-2RS (5'-AATCGCTCAACAAAGCAGATGAGT-3') primer and for hotspot-2 of *FKS2* by using CgFKS2-2FS (5'-AGGTACACAATTGCCCGTAGA-3') or CgFKS2-2RS (5'-TGTCACTCAATAGAGCAGCAGAA-3') primer. Sequencing reactions were performed and processed as described previously^24^. Sequence data for hotspot-1 and hotspot-2 regions of *FKS1* and *FKS2* were compared with corresponding sequences from reference *C. glabrata* strain ATCC90030 by using Clustal Omega (https://www.ebi.ac.uk/Tools/msa/clustalo/).

### PCR-sequencing of *ERG11* gene

The *ERG11* gene was amplified as two overlapping fragments by using *C. glabrata* ATCC90030 as reference. The N-terminal fragment was amplified by using CgERG11F (5'-TCCACCTCGAACCCGTATA-3') and CgERG11RS3 (5'-ATCAAGACACCAATCAATAGGTT-3') primers while C-terminal fragment was amplified by using CgERG11FS3 (5'-GACGTGAGAAGAACGATATCCA-3') and CgERG11R (5'-TCCATGTTGATATTCACGATGACT-3') primers. Other PCR reaction and cycling conditions were same as described previously^28,29^. N-terminal amplicons were sequenced with CgERG11FS1 (5'-GAACCCGTATACTCATCTCGTA-3'), CgERG11FS2 (5'-GGTGATATCTTCTCTTTCATGCTA-3'), CgERG11RS3 (5'- AGTAAGCAGCTTCAGCGGAAACA-3') and CgERG11RS4 (5'-ATCAAGACACCAATCAATAGGTT-3') primers. C-terminal amplicons were sequenced with CgERG11FS3 (5'-GACGTGAGAAGAACGATATCCA-3'), CgERG11FS4 (5'-GTTACACTCACTTGCAAGAAGAA-3'), CgERG11RS1 (5'-CACGATGACTTACTATTAGGCTAA-3') and CgERG11RS2 (5'-CGAAACCGTAATCAACTTCGTCA-3') primers. Sequencing reactions were performed and processed as described previously^28,29^. Nucleotide and amino acid sequences were compared with wild-type sequence from *C. glabrata* ATCC90030 using Clustal Omega (http://www.ebi.ac.uk/Tools/msa/clustalo/).

The DNA sequence data have been submitted to GenBank/EMBL/DDBJ databases under accession numbers LR757901 to LR757940.

### Molecular fingerprinting of micafungin-resistant *C. glabrata* isolates

The phylogenetic relationship among micafungin-resistant and some randomly selected micafungin-susceptible *C. glabrata* isolates was also studied. The DNA sequence data for ITS region of rDNA together with hotspot-1 and hotspot-2 of *FKS1* and hotspot-1 and hotspot-2 of *FKS2* were concatenated and the combined data set were used to construct Neighbor-Joining phylogenetic tree using Maximum Composite Likelihood settings by using Molecular Evolutionary Genetics Analysis Version 7.0 (MEGA7) software (http://www.megasoftware.net/mega.php). The robustness of tree branches was assessed by bootstrap analysis of 1,000 replicates. The isolates were considered belonging to the same genotype when they contained the same sequence for all loci.

### Molecular fingerprinting of fluconazole-resistant *C. glabrata* isolates

The phylogenetic relationship among fluconazole-resistant and some randomly selected fluconazole-susceptible *C. glabrata* isolates was studied by constructing dendrograms based on DNA sequence data for *ERG11* alone or in combination with ITS region of rDNA and hotspot-1 and hotspot-2 of *FKS1* and *FKS2* genes. The sequences were concatenated and the combined sequence data set were used to construct phylogenetic tree and the data were interpreted as described above.

## Supplementary information

Supplementary Information.
