# Supplementary material for: Antifungal drug susceptibility, molecular basis of resistance to echinocandins and molecular epidemiology of fluconazole resistance among clinical Candida glabrata isolates in Kuwait
Source: Sci Rep. 2020 Apr 10;10:6238. doi: 10.1038/s41598-020-63240-z (PMC7148369; doi:10.1038/s41598-020-63240-z)
Supplement: Supplementary file 1 — Supplementary Information. [file 41598_2020_63240_MOESM1_ESM.docx]

**Antifungal drug susceptibility, molecular basis of resistance to echinocandins and molecular epidemiology of fluconazole resistance among clinical *Candida glabrata* isolates in Kuwait**

**Authors:**

**1. Zahraa F. Al-Baqsami**, Department of Microbiology, Faculty of Medicine, Kuwait University, Jabriya, Kuwait. [zfba_kw@hotmail.com](mailto:zfba_kw@hotmail.com)
**2. Suhail Ahmad**, Department of Microbiology, Faculty of Medicine, Kuwait University, Jabriya, Kuwait. [suhail_ah@hsc.edu.kw](mailto:suhail_ah@HSC.EDU.KW)

**3. Ziauddin Khan**, Department of Microbiology, Faculty of Medicine, Kuwait University, Jabriya, Kuwait. [ziauddin381944@gmail.com](mailto:ziauddin381944@gmail.com)

**S1 Table. Summary of susceptibility test results for micafungin by Etest and broth microdilution (BMD) method and for caspofungin by Etest together with data for hotspot-1 and hotspot-2 of *FKS1* and *FKS2* genes for 75 *C. glabrata* isolates from Kuwait**

| **Serial no.** | **Isolate No.** | **Clinical source^a^** | **Micafungin^b^** | | | **Caspofungin^c^** | | ***FKS* genotypes^d^** | | | |
| --- | --- | --- | --- | --- | --- | --- | --- | --- | --- | --- | --- |
|  |  |  |  |  |  |  |  | ***FKS1*** | | ***FKS2*** | |
|  |  |  | **Etest MIC (µg/ml)** | **EUCAST BMD MIC (µg/ml)** | **Category** | **Etest MIC (µg/ml)** | **Category** | **HS-1*** | **HS-2** | **HS-1** | **HS-2** |
| **1** | Kw3148/07 | Sputum | 0.016 | 0.007 | S | 0.094 | S | wt | wt | wt | wt |
| **2** | Kw123/08 | Sputum | 0.016 | 0.015 | S | 0.012 | S | wt | wt | wt | wt |
| **3** | Kw948/08 | Urine | 0.008 | ≤0.003 | S | 0.094 | S | wt | wt | wt | wt |
| **4** | Kw2717/8 | Blood | 0.012 | 0.015 | S | 0.064 | S | wt | wt | wt | wt |
| **5** | Kw210/19 | Blood | 0.012 | 0.007 | S | 0.064 | S | wt | wt | wt | wt |
| **6** | Kw583/09 | Blood | 0.012 | ≤0.003 | S | 0.032 | S | wt | wt | wt | wt |
| **7** | Kw710/09 | Sputum | 0.012 | 0.007 | S | 0.023 | S | wt | wt | wt | wt |
| **8** | Kw3025/09 | Urine | 0.016 | ≤0.003 | S | 0.023 | S | wt | wt | wt | wt |
| **9** | Kw105/13 | ET secretion | 0.008 | ≤0.003 | S | 0.094 | S | wt | wt | wt | wt |
| **10** | Kw567/14 | Urine | 0.023 | ≤0.003 | S | 0.125 | S | wt | wt | wt | wt |
| **11** | Kw632/14 | Blood | 0.012 | 0.015 | S | 0.125 | S | wt | wt | wt | wt |
| **12** | Kw1892/14 | Skin | 0.012 | 0.007 | S | 0.094 | S | *F625F* | wt | wt | wt |
| **13** | Kw2035/14 | Rectal swab | 0.012 | 0.007 | S | 0.25 | I | wt | wt | wt | wt |
| **14** | Kw2059/14 | Sputum | 0.016 | 0.007 | S | 0.25 | I | wt | wt | wt | wt |
| **15** | Kw2111/14 | Urine | 0.008 | 0.007 | S | 0.25 | I | wt | wt | wt | wt |
| **16** | Kw2112/14 | BAL | 0.016 | 0.007 | S | 0.25 | I | wt | wt | wt | wt |
| **17** | Kw2188/14 | Blood | 0.012 | 0.007 | S | 0.25 | I | wt | wt | wt | wt |
| **18** | Kw2214/14 | ET secretion | 0.016 | 0.007 | S | 0.25 | I | wt | wt | wt | wt |
| **19** | Kw2221/14 | Urine | 0.016 | 0.007 | S | 0.38 | I | wt | wt | wt | wt |
| **20** | Kw2231/14 | Urine | 0.016 | 0.007 | S | 0.25 | I | wt | wt | wt | wt |
| **21** | Kw3786/14 | Urine | 0.023 | 0.007 | S | 0.25 | I | wt | wt | wt | wt |
| **22** | Kw21/15 | BAL | 0.016 | 0.007 | S | 0.38 | I | *F625F* | wt | wt | wt |
| **23** | Kw96/15 | Urine | 0.016 | 0.007 | S | 0.094 | S | wt | wt | wt | wt |
| **24** | Kw131/15 | Sputum | 0.016 | ≤0.003 | S | 0.38 | I | *F625F* | wt | wt | wt |
| **25** | **Kw164/15** | **Urine** | **0.125** | **2** | **R** | **0.75** | **R** | ***F625F*** | **wt** | ***S663P*** | **wt** |
| **26** | Kw185/15 | Urine | 0.023 | 0.007 | S | 0.25 | I | wt | wt | wt | wt |
| **27** | Kw330/15 | Tracheal secretion | 0.008 | ≤0.003 | S | 0.5 | R | wt | wt | wt | wt |
| **28** | Kw383/15 | Ascitic fluid | 0.016 | 0.007 | S | 0.19 | S | wt | wt | wt | wt |
| **29** | Kw392/15 | Urine | 0.016 | 0.007 | S | 0.25 | I | wt | wt | wt | wt |
| **30** | Kw467/15 | Bed sore | 0.012 | 0.007 | S | 0.38 | I | wt | wt | wt | wt |
| **31** | Kw480/15 | Pus | 0.006 | ≤0.003 | S | 0.38 | I | wt | wt | wt | wt |
| **32** | Kw590/15 | Sputum | 0.008 | ≤0.003 | S | 0.125 | S | wt | wt | wt | wt |
| **33** | Kw945/15 | Cavity fluid | 0.016 | 0.007 | S | 0.25 | I | *F625F* | wt | wt | wt |
| **34** | Kw1257/15 | Skin | 0.016 | 0.03 | S | 0.19 | S | wt | wt | wt | wt |
| **35** | Kw1302/15 | Vulva swab | 0.023 | 0.007 | S | 0.094 | S | wt | wt | wt | wt |
| **36** | Kw1348/15 | Urine | 0.012 | 0.007 | S | 0.125 | S | wt | wt | wt | wt |
| **37** | Kw1804/15 | HV swab | 0.016 | 0.007 | S | 0.19 | S | wt | wt | wt | wt |
| **38** | Kw1856/15 | Urine | 0.008 | 0.007 | S | 0.094 | S | wt | wt | wt | wt |
| **39** | Kw2098/15 | Sputum | 0.016 | ≤0.003 | S | 0.25 | I | wt | wt | wt | wt |
| **40** | Kw2516/15 | Urine | 0.016 | 0.007 | S | 0.19 | S | wt | wt | wt | wt |
| **41** | Kw2591/15 | Blood | 0.016 | 0.007 | S | 0.19 | S | wt | wt | wt | wt |
| **42** | Kw2691/15 | Tracheal secretion | 0.023 | 0.007 | S | 0.032 | S | wt | wt | wt | wt |
| **43** | Kw2813/15 | Urine | 0.012 | 0.007 | S | 0.032 | S | wt | wt | wt | wt |
| **44** | Kw3060/15 | Wound swab | 0.016 | 0.015 | S | 0.094 | S | wt | wt | wt | wt |
| **45** | **Kw3646/15** | **Tracheal secretion** | **0.125** | **1** | **R** | **0.38** | **I** | **wt** | **wt** | ***S663P*** | **wt** |
| **46** | Kw79/16 | Urine | 0.008 | 0.007 | S | 0.094 | S | wt | wt | wt | wt |
| **47** | Kw102/16 | Urine | 0.012 | 0.007 | S | 0.19 | S | wt | wt | wt | wt |
| **48** | Kw381/16 | Wound swab | 0.023 | 0.007 | S | 0.094 | S | wt | wt | wt | wt |
| **49** | Kw442/16 | Urine | 0.016 | 0.007 | S | 0.19 | S | wt | wt | wt | wt |
| **50** | **Kw458/16** | **Urine** | **0.25** | **0.25** | **R** | **0.38** | **I** | **wt** | **wt** | ***∆F659*** | **wt** |
| **51** | Kw515/16 | Blood | 0.008 | 0.007 | S | 0.19 | S | *F625F* | wt | wt | wt |
| **52** | Kw647/16 | Urine | 0.016 | 0.007 | S | 0.25 | I | wt | wt | wt | wt |
| **53** | Kw724/16 | Blood | 0.023 | 0.007 | S | 0.023 | S | wt | wt | wt | wt |
| **54** | Kw752/16 | Blood | 0.032 | 0.007 | S | 0.125 | S | wt | wt | wt | wt |
| **55** | Kw1140/16 | Sputum | 0.012 | 0.03 | S | 0.38 | I | wt | wt | wt | wt |
| **56** | Kw1226/16 | PEG site swab | 0.016 | 0.007 | S | 0.19 | S | wt | wt | wt | wt |
| **57** | Kw1323/16 | NA | 0.012 | 0.007 | S | 0.38 | I | *F625F* | wt | wt | wt |
| **58** | Kw1567/16 | Urine | 0.016 | 0.007 | S | 0.047 | S | wt | wt | wt | wt |
| **59** | Kw1697/16 | Wound swab | 0.008 | 0.007 | S | 0.125 | S | wt | wt | wt | wt |
| **60** | Kw1830/16 | ET secretion | 0.023 | 0.007 | S | 0.19 | S | wt | wt | wt | wt |
| **61** | Kw1921/16 | BAL | 0.012 | 0.007 | S | 0.032 | S | wt | wt | wt | wt |
| **62** | Kw1959/16 | Urine | 0.016 | 0.007 | S | 0.008 | S | wt | wt | wt | wt |
| **63** | Kw2432/16 | Cavity fluid | 0.023 | 0.007 | S | 0.25 | I | wt | wt | wt | wt |
| **64** | **Kw3554/16** | **Urine** | **0.125** | **2** | **R** | **0.75** | **R** | **wt** | **wt** | ***S663P*** | **wt** |
| **65** | Kw336/17 | ET secretion | 0.004 | ≤0.003 | S | 0.38 | I | wt | wt | wt | wt |
| **66** | Kw512/17 | Urine | 0.012 | ≤0.003 | S | 0.094 | S | wt | wt | wt | wt |
| **67** | Kw620/17 | ET secretion | 0.016 | 0.007 | S | 0.19 | S | wt | wt | wt | wt |
| **68** | Kw781/17 | Urine | 0.032 | 0.007 | S | 0.19 | S | wt | wt | wt | wt |
| **69** | Kw1132/17 | PD | 0.016 | 0.015 | S | 0.19 | S | wt | wt | wt | wt |
| **70** | Kw1171/17 | Blood | 0.023 | 0.007 | S | 0.094 | S | wt | wt | wt | wt |
| **71** | Kw1363/17 | Urine | 0.016 | 0.015 | S | 0.047 | S | *F625F* | wt | wt | wt |
| **72** | Kw1500/17 | Blood | 0.008 | 0.007 | S | 0.094 | S | *F625F* | wt | wt | wt |
| **73** | Kw1721/17 | Blood | 0.008 | 0.007 | S | 0.016 | S | wt | wt | wt | wt |
| **74** | Kw1807/17 | Tracheal secretion | 0.023 | 0.015 | S | 0.125 | S | wt | wt | wt | wt |
| **75** | **Kw2138/17** | **Urine** | **0.125** | **2** | **R** | **0.75** | **R** | **wt** | **wt** | ***S663P*** | **wt** |

^a^ET, endotracheal; BAL, bronchoalveolar lavage; HV, high vaginal; PD, peritoneal dialysis; PEG, percutaneous endoscopic gastrostomy; NA, not available; ^b^Susceptibility data interpretation by using EUCAST breakpoints; ^c^Susceptibility data interpretation by using CLSI breakpoints; S, susceptible; I, intermediate; R, resistant

^d^HS-1, hotspot-1; HS-2, hotspot-2; wt, wild type; *Synonymous hotspot-1 *FKS1* mutations; Isolates with nonsynonymous mutations in hotspot-1 of *FKS2* are highlighted in bold.
